# From Biopsy to Diagnosis: Navigating Aggressive B-Cell Lymphomas in Practice

**DOI:** 10.3390/medicina61050842

**Published:** 2025-05-02

**Authors:** Georgian Halcu, Anca Evsei-Seceleanu, Mihai Cerbu, Marina Alina Bara, Andrei Turbatu, Mihail Constantin Ceausu

**Affiliations:** 1 Anatomical Pathology Department, Faculty of General Medicine, Universitatea de Medicina si Farmacie Carol Davila, Bulevardul Eroii Sanitari 8, 050474 București, Romania; anca.evsei@umfcd.ro (A.E.-S.);; 2 Spitalul Clinic Coltea, Bulevardul Ion C. Brătianu 1, 030167 București, Romania; 3 Spitalul Clinic Sfanta Maria, Bulevardul Ion Mihalache 37-39, 011172 București, Romania; 4 Spitalul Universitar de Urgenta, Splaiul Independenței 169, 050098 București, Romania; 5 Institutul National de Endocrinologie C. I. Parhon, B–dul Aviatorilor, nr. 34–38, 011863 Bucureşti, Romania

**Keywords:** pathology, non-Hodgkin lymphoma, DLBCL, immunohistochemistry, hematology

## Abstract

Diffuse large B-cell lymphoma (DLBCL), recognized as the most prevalent variant of adult non-Hodgkin lymphoma, presents considerable challenges in diagnosis owing to its diverse morphological features and frequent extranodal involvement, which may frequently mimic nonhematopoietic neoplasms. The 2022 WHO Classification of Lymphoid and Hematopoietic Tissues provides essential updates, highlighting the necessity of combining morphology, immunohistochemistry, cytogenetics, and molecular testing for precise subclassification. This review presents a practical method for differentiating DLBCL from other aggressive B-cell neoplasms, such as Burkitt lymphoma, B-lymphoblastic lymphoma, and mantle cell lymphoma. It highlights vital diagnostic tools, including CD45, B/T-cell markers, germinal center markers, and the Hans algorithm, as well as the role of FISH in identifying rearrangements of key genes MYC, BCL2, and BCL6, which are significant for recognizing double-hit and triple-hit lymphomas. Special focus is given to EBV-associated DLBCL and uncommon subtypes featuring plasmablastic or ALK-positive traits. This review aims to enhance diagnostic accuracy and ensure appropriate treatment strategies for patients with large B-cell lymphomas by emphasizing thorough morphological evaluation, specific adjunct testing, and adherence to the most recent classification standards.

## 1. Introduction

The WHO 2022 Classification of Lymphoid and Hematopoietic Tissues edition addresses large B-cell neoplasms, mainly diffuse large B-cell lymphomas (DLBCLs), accounting for 30% to 40% of adult non-Hodgkin lymphoma instances [1,2].

These neoplasms can be challenging for pathologists in general surgical pathology as they often manifest as extranodal lesions; hence, their clinical and radiological features may resemble those of other nonhematopoietic tumors. The morphology of these lymphoid lesions tends to be poorly differentiated, resembling undifferentiated carcinomas, melanomas, or sarcomas.

Occasionally, these aggressive B-cell lymphomas are subject to unnecessary surgeries or improper chemotherapy. Yet, it is essential to recognize the more favorable prognosis for patients with large B-cell lymphomas. Current treatment protocols can yield over 50% long-term survival rates for those affected by this disease [3,4,5,6,7].

It is essential to differentiate typical DLBCL NOS cases from other aggressive B-cell lymphomas that exhibit comparable characteristics, which adds complexity diagnosis. The latest 2022 WHO Classification of Lymphoid and Hematopoietic Tissues provides significant updates for these conditions [8,9,10,11].

Given that DLBCL is the predominant entity, our focus will be on it while also addressing other types that require differentiation. As such, DLBCL NOS is sometimes viewed as an exclusion diagnosis. Distinguishing it from other aggressive B-cell neoplasms is crucial since certain forms, like B-lymphoblastic lymphoma, Burkitt lymphoma, and DLBCL NOS, have favorable prognoses with appropriate treatment regimens.

## 2. Lymphoma, Carcinoma, Melanoma, or Sarcoma—What Is the Difference?

**Figure 1:**
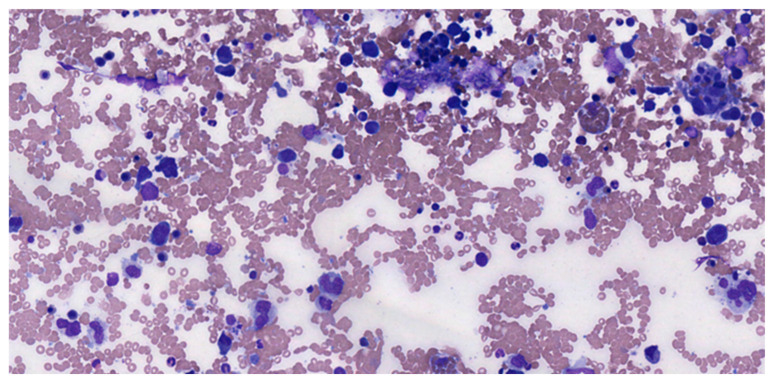
DLBCL pleural effusion MGG 20×.

The initial procedure involves a morphological assessment to distinguish between lymphoid neoplasia and solid tumors (such as melanoma, sarcoma, or undifferentiated carcinoma). In addition to the frozen sections of suspected lymphoma samples, a few touch imprints should be prepared (Figure 1), as this allows for a clearer view of lymphoid morphology and facilitates a more thorough cytomorphologic analysis than what a frozen section can provide.

Lymphoid lesions present individual cells or clusters of cells distributed on the slide, unlike solid tumors, which form dense aggregates [12,13,14] When touch imprints show lymphoid characteristics, it is important to send a part of the sample for flow cytometry or cytogenetics. Where there are small biopsy fragments, it is best to prioritize getting a permanent section, as paraffin-embedded samples allow for future immunohistochemical analyses. Thus, it is essential to keep some material for histology rather than submitting the entire lymphoid sample for flow cytometry [15,16].

Cytogenetic tests are warranted in lymphoid lesions, particularly when lymphoma is strongly suspected. Fluorescence in situ hybridization (FISH) can still be performed on formalin-fixed paraffin-embedded specimens.

Following histological examination, immunohistochemical panels are employed to assess the lesion. It is crucial to perform CD45 (LCA) IHC when lymphoma is a possibility. Caution should be exercised with lesions that might be CD45 negative but retain a lymphoid appearance; targeted T- and B-cell stains should be conducted in such instances.

## 3. B-Cell Lymphoma, T-Cell Lymphoma, or Hodgkin Lymphoma

B-lineage or B-cell differentiation can be established through flow cytometry or IHC methods. For suspected B-cell neoplasia, it is essential to utilize a marker for T lymphocytes alongside at least one B-cell stain for assessment. CD20 (B marker) and CD3 (T marker) are commonly employed markers, but since certain B-cell lymphomas may test negative for CD20, incorporating an additional B-cell marker is crucial to ensure no lesions are overlooked.

CD20, a pan-B-cell marker, is typically present in mature B-cell neoplasms. Its loss may suggest a more differentiated or advanced disease state, often with plasmablastic features, where neoplastic cells undergo terminal differentiation, leading to reduced CD20 expression [17,18]. This creates a diagnostic challenge, as traditional markers may not identify the B-cell lineage. Thus, including alternative B-cell markers (CD79a and PAX5) is crucial for confirming lineage and ensuring accurate diagnosis.

Research indicates that in cases of CD20-negative diffuse large B-cell lymphoma (DLBCL), the inclusion of CD79a and PAX5 in the immunohistochemical panel can significantly enhance diagnostic specificity and sensitivity [19]. Studies have shown that when CD20 is lost, the retention of other B-cell markers such as CD19, CD79a, and PAX5 can still indicate the underlying B-cell malignancy and guide appropriate therapeutic decisions, particularly in cases like DLBCL, where treatment outcomes may vary based on the lineage determined [20].

Moreover, retaining the ability to identify the B-cell lineage through markers such as CD79a and PAX5 enables more accurate diagnosis and classification, which is crucial for planning effective management strategies, particularly for treatment regimens that may involve targeted therapies. Thus, in clinical practice, it is essential to employ a comprehensive immunophenotypic panel that includes these additional markers to accurately characterize B-cell neoplasms, especially when conventional markers like CD20 are not expressed.

Specifically, plasmablastic lymphoma and ALK+ large B-cell lymphomas often present as CD20-negative. In such cases, markers like PAX5 and/or CD79a can be used for any lymphoma exhibiting Hodgkin or non-Hodgkin lymphoma features.

## 4. Histological Characteristics and Developmental Patterns

Once the B-lineage is confirmed, it is crucial to assess the growth pattern and determine whether the architecture is follicular/nodular or diffuse. Generally, a nodular structure indicates a potential for follicular lymphoma. Grade 3 follicular lymphoma features many large centroblasts, which may resemble DLBCL NOS [21,22]. Encountering both grade 3 follicular lymphoma and DLBCL in the same specimen is not unusual, as this transformation is recognized to occur.

In some cases, lymph nodes with nodules or diffuse regions of large, immature cells in the head and neck of a pediatric or young adult patient can suggest large B-cell lymphoma associated with IRF4 translocation [23]. Assessing nodular or follicular architecture can sometimes be complicated; however, specific immunohistochemical stains can be beneficial. Follicular dendritic markers, such as CD21 and CD23, are practical, while CD10, BCL6, BCL2, and Ki-67 help identify the germinal center phenotype in follicular or diffuse lymphomas [24,25,26].

## 5. Morphological Evaluation

Large cells are defined by having nuclei that are either comparable in size to normal histiocytes or at least twice the size of a normal lymphocyte [27]. In practice, a more reliable method for assessing cell size is to compare it to the red blood cells typically seen in tissue samples. Therefore, if the nucleus of a lymphoid cell can be estimated to accommodate two or more red blood cells, it suggests the presence of a large lymphoma cell [28].

**Figure 2:**
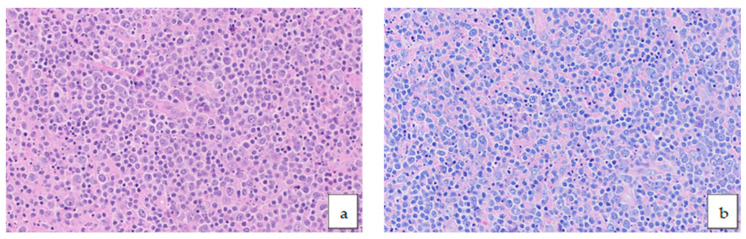
DLBCL morphology HE 40× ((**a**), left) and Giemsa 40× ((**b**), right).

Cell size is a critical factor in classifying B-cell lymphomas. The majority of DLBCL NOS cases feature medium to large cells, with most qualifying as the centroblastic subtype (Figure 2a,b). As a practical tip, reducing the light intensity of the optical microscope’s condenser may help reveal subtle follicular architecture when immunohistochemical stains are unavailable.

Medium-sized lymphoma cells of B origin comprise mantle cell lymphoma (MCL), Burkitt lymphoma (BL), and B-lymphoblastic lymphoma (B-LBL) [29]. Each can display variable immature blastic features. Burkitt lymphoma is composed of monotonous, medium-sized cells; accordingly, the presence of any population of large cells is inconsistent with BL [29,30,31]. In the differential diagnosis, MCL and B-LBL should be considered when dealing with a medium-sized B-cell population.

DLBCL cases exhibit variable morphology, with cells showing considerable differences in size and shape. These lymphoid neoplasms possess irregular nuclei, demonstrating significant variability in dimensions and form. In contrast, BL features a consistent population, exhibiting mildly angulated borders that align with adjacent cells. This results in a tile-like look alongside a starry sky appearence and uniformly spread tangible body macrophages. B-LBL constitutes a significant variant of high-grade B-cell lymphoma, distinguished by its unique blastic characteristics. It is imperative to differentiate it from diffuse large B-cell lymphoma (DLBCL) and Burkitt lymphoma (BL), both of which are markedly aggressive neoplasms. These conditions frequently affect pediatric populations; however, it is encouraging to note that they possess the potential for curability.

Cases of B-LBL feature monomorphous immature cells, ranging in size from small to large, with finely dispersed chromatin. Ancillary tests such as flow cytometry or IHC are essential for distinguishing between B-LBL and BL [32,33,34]. Burkitt lymphoma typically tests positive for CD10 while usually testing negative for BCL2, TdT, and CD34. In contrast, B-LBLs express CD10, CD34, TdT, or BCL2 but lack surface light chain expression. It is crucial to differentiate between DLBCL and BL, as these two malignancies undergo distinct treatment regimens that, when properly administered, are highly effective.

Occasionally, these two B-cell lymphomas present with similar morphological features and overlapping characteristics, with many being classified as either high-grade B-cell lymphomas with MYC and BCL2 and/or BCL6 rearrangements (HGBCL-DH or HGBL-TH) or as high-grade B-cell lymphomas, not otherwise specified (HGBCL, NOS). Therefore, performing FISH for MYC, BCL2, and BCL6 rearrangements is essential to differentiate DH or TH lymphomas.

The latest WHO classification introduces two categories: HGBCL with MYC and BCL2 and/or BCL6 rearrangements, and HGBCL NOS. These replace the earlier unclassifiable B-cell lymphoma, which displayed characteristics of both DLBCL and BL.

## 6. Medium-Sized B-Cell Lymphoma with CCND1 Translocation

Excluding mantle cell lymphoma’s blastic or pleomorphic variant is a significant concern in high-grade B-cell lymphomas. The blastoid type is distinct from traditional MCL in that its cells exhibit a resemblance to lymphoblasts, characterized by a more dispersed chromatin structure and a significant number of mitotic figures. Conversely, the pleomorphic variant is distinguished by atypical cells, many of which are notable for their larger size, irregular nuclear shapes, pale cytoplasm, and prominent nucleoli.

Mantle cell lymphoma (MCL) is marked by the chromosomal rearrangement t(11;14)(q13;q32), which juxtaposes the cyclin D1 gene (CCND1) on chromosome 11 with the immunoglobulin heavy chain locus on chromosome 14. Overexpression of cyclin D1 drives MCL by promoting uncontrolled cell cycle progression and contributing to its aggressive behavior [35,36]. Immunohistochemistry for cyclin D1 is a common method to assist in diagnosing MCL. Positive cyclin D1 staining, seen as nuclear overexpression, is associated with the t(11;14) translocation [37]. Most MCL cases show strong cyclin D1 positivity, a key feature that helps differentiate MCL from other B-cell neoplasms without such expression. Several methods can be used to detect this chromosomal translocation and the associated overexpression of cyclin D1. Fluorescence in situ hybridization is a sensitive technique for visualizing specific chromosomal rearrangements, like t(11;14). Pathologists determine this translocation in lymphoid cells using probes for the CCND1 gene and the immunoglobulin heavy chain locus. FISH analysis provides evidence of the genetic alteration crucial for diagnosing MCL [38].

Regardless of CD5 expression, all medium- to large-cell B neoplasms should be screened using cyclin D1. The rearrangement of CCND1. In rare instances of cyclin D1-negative MCL, immunohistochemistry is aided by another stain, which is particularly useful—SOX11.

Approximately 10% of DLBCL may express CD5 either focally or diffusely, leading to confusion with MCL.

Unfortunately, these lymphomas are unrelated to MCL and are associated with high-risk clinical characteristics. These cases do not express cyclin D1 or SOX11, and FISH for CCND1 translocation yields a negative result.

## 7. Germinal Center Phenotype

To identify any B-cell lymphoma, CD10 (CALLA) expression must be assessed using flow cytometry or IHC. This assessment is essential for distinguishing between different types of B-cell lymphomas. Notably, CD10 and BCL6 serve as germinal center markers present in Burkitt lymphoma (BL) cases. Since these markers are also found in diffuse large B-cell lymphoma (DLBCL), the absence of CD10 and BCL6 can effectively rule out BL. Another argument for conducting a CD10 stain in cases of diffuse large B-cell lymphoma (DLBCL) is the utilization of the Hans algorithm.

CD10 and BCL6 are usually expressed in GCB (germinal center B-cell) patients. CD10 serves as a marker for germinal center B-cells, whereas BCL6 acts as a transcriptional repressor essential for the formation and function of germinal centers. Within the DLBCL context, the expression of both CD10 and BCL6 typically suggests a GCB (germinal center B-cell-like) subtype. If a tumor displays positivity for both markers, it is more likely categorized as GCB [39,40]. On the other hand, MUM1 (also known as IRF4), which is found in activated B-cells, often correlates positively with the ABC subtype. In a considerable number of DLBCLs, MUM1 positivity appears alongside the absence of CD10 and BCL6 expression, indicating a shift from germinal center to post-germinal center stages, which is typical of the ABC subtype [41,42].

Should the results for CD10 and BCL6 remain inconclusive, it may be advantageous to perform staining for BCL2, given that Burkitt lymphomas infrequently express this marker; however, it is noteworthy that certain DLBCL cases may exhibit variable expression of BCL2. Burkitt lymphomas are known to be positive for c-MYC in nearly all cases, whereas only a tiny number of DLBCLs express this marker.

## 8. Hans Algorithm—For CD10-DLBCL, Request BCL6 and MUM1 IHC

Once grade 3 FL, BL, MCL, and B-LBL are excluded based on morphological and immunohistochemical criteria, a few additional stains are required to implement the Hans algorithm for a comprehensive evaluation of DLBCL. This algorithm provides prognostic insights that distinguish between the germinal center and activated B-cell subtypes of DLBCL. While practical, the immunohistochemical method for this classification has a 20% error rate.

**Figure 3:**
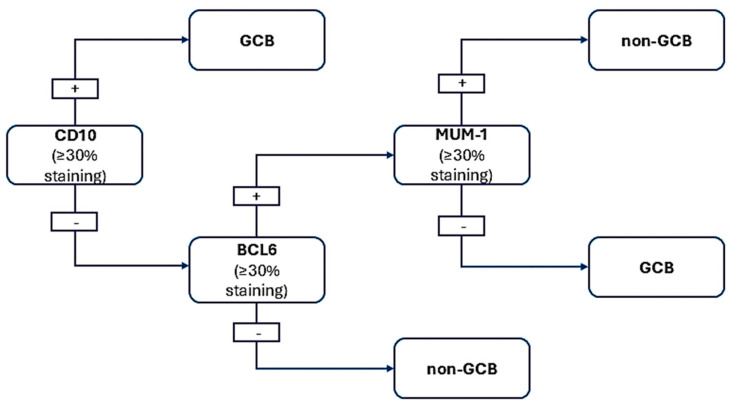
Hans algorithm for DLBCL.

The Hans algorithm (Figure 3) utilizes IHC staining for CD10, BCL6, and MUM1/IRF4 to identify germinal center or activated B-cell phenotypes. The positivity threshold for the markers used—CD10, BCL6, and MUM1—is generally set at 30%. For a lymphoid proliferation to be classified as expressing a specific marker, at least 30% of the cells must show positive staining [43,44]. This criterion has been validated in numerous studies, demonstrating that such thresholds are essential for consistent classification and prognostic stratification in cases of DLBCL [45,46]. Around 40% of DLBCL cases show CD10 expression. For cases that are CD10-negative, testing for BCL6 and MUM1 is necessary, as nearly 75% and 50% of DLBCL cases test positive for BCL6 and MUM1, respectively.

## 9. Epstein–Barr Virus Infection and Large B-Cell Lymphomas

**Figure 4:**
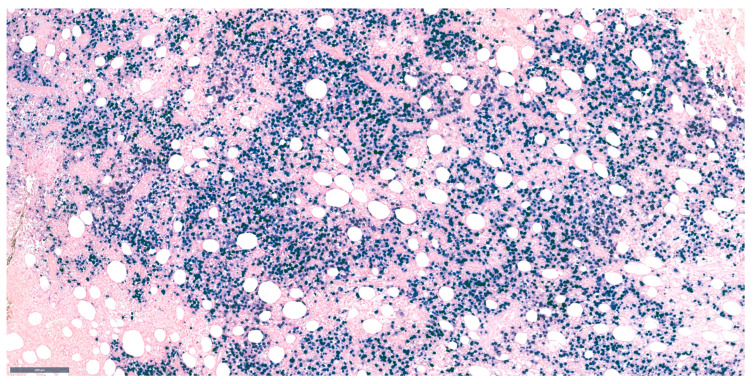
EBER ISH in an EBV-positive DLBCL 10×.

Epstein–Barr virus (EBV) is known to be associated with lymphoid malignancies, and all large B-cell lymphomas should undergo testing with in situ hybridization for EBV–encoded RNA (EBER) (Figure 4). This subtype of lymphoma, known as EBV-positive DLBCL, is uncommon and primarily impacts individuals over 50 years of age. Its pathogenesis is most likely related to age-related immunosuppression [47].

This disease is often found more in Asian and Latin American populations. Its morphological features include immunoblastic cells and cells similar to Hodgkin–Reed–Sternberg. CD30 expression is common in these lymphomas, requiring differentiation from lymphomatoid granulomatosis and EBV-positive mucocutaneous ulcers [48,49,50,51,52].

## 10. BCL2 and C-Myc Ihc for Double Expresser DLBCL

In cases of DLBCL, immunohistochemical staining for BCL2 and c-MYC identifies “double expresser” lymphomas, a subgroup associated with a poor prognosis, though not as severe as double-hit lymphomas [53,54]. While most double-hit lymphomas show positivity for MYC and BCL2 proteins and qualify as double-expresser DLBCL, many double-expresser DLBCL cases do not fall under the DH category. To qualify as double-expresser DLBCL, over 50% of cells must test positive for BCL2 and at least 40% for MYC via IHC.

## 11. Cytogenetic Testing for Double-Hit/Triple-Hit B-Cell Lymphoma

FISH testing is necessary for all large B-cell lymphomas to assess the presence of double-hit or triple-hit lymphoma (high-grade B-cell lymphoma featuring MYC and BCL2 and/or BCL6 rearrangements). High-grade B-cell lymphomas categorized as double-hit (HGBCL-DH) or triple-hit (HGBCL-TH) are associated with unfavorable outcomes when treated with R-CHOP, thereby warranting the consideration of more intensive therapeutic regimens. Moreover, there exists an increased risk of central nervous system involvement, necessitating the implementation of appropriate prophylactic measures.

To confirm a diagnosis of high-grade B-cell lymphoma with double-hit (DH) or triple-hit (TH) features (HGBCL-DH/TH), the specific cytogenetic findings involve the rearrangement of key oncogenes, notably MYC, BCL2, and BCL6. These genetic alterations indicate a complex biological behavior and a more aggressive disease profile than typical high-grade B-cell lymphomas. The rearrangement of the MYC oncogene is a definitive cytogenetic finding in both double-hit and triple-hit lymphomas. This rearrangement is pivotal as it is associated with increased cell proliferation and poor prognosis [55].

In double-hit lymphoma (DHL), there are concurrent MYC and BCL2 (or BCL6) rearrangements [56]. Specifically, the presence of a MYC rearrangement alongside a BCL2 rearrangement qualifies the diagnosis as double-hit lymphoma.

In triple-hit lymphoma (THL), the diagnosis is confirmed with the presence of MYC alongside both BCL2 and BCL6 rearrangements. This indicates a more complex pathology and an even more aggressive clinical course than double-hit lymphoma [57]. These rearrangements are typically confirmed using FISH analysis, which allows for visualization of these chromosomal alterations at the cellular level. FISH is the gold standard for diagnosing these high-grade B-cell lymphomas, particularly because conventional cytogenetic techniques may not always detect these rearrangements [58]. However, this technique is costly. A more economical strategy involves evaluating all cases for MYC rearrangements, followed by an assessment of BCL2 (and BCL6) along with the MYC translocation partner. Amplification, copy number increases, or somatic mutations detected by FISH in the absence of a rearrangement, by definition, exclude HGBCL-DH/TH. Karyotypes or FISH that exhibit more than three chromosomal abnormalities rule out BL. Approximately 10% of BLs do not demonstrate a MYC rearrangement, yet a BL diagnosis remains feasible with classic morphology and immunohistochemistry.

Additionally, a subset of B-cell lymphomas that histologically resemble BL do not have the MYC rearrangement but possess 11q aberrations. In the 2022 version of the WHO classification, these cases are categorized as high-grade B-cell lymphomas featuring 11q aberration.

## 12. DLBCL Proliferation Index

The proliferation index is assessed using immunohistochemistry and the Ki67 marker. Nearly all Burkitt lymphoma (BL) cases exhibit almost 100% Ki67 positivity; therefore, any B-cell lymphoma with a proliferation index below 90% is less likely to be classified as BL. B-lymphoblastic lymphoma (B-LBL) also demonstrates a high proliferation rate but can be differentiated by its morphology and immunophenotypic characteristics.

This high proliferation rate correlates with the disease’s aggressive nature, classifying it as a high-grade lymphoma [59]. Such a high threshold is based on the biology of Burkitt lymphoma, characterized by rapid cell turnover and high cellular proliferation, driven by MYC oncogene overexpression [60].

A lower Ki-67 index is not a strong indicator of Burkitt lymphoma. The Ki-67 index typically ranges from 70% to 80% for non-Burkitt high-grade B-cell lymphomas. This range indicates aggressive disease, but not the extreme proliferation seen in Burkitt lymphoma [21,61]. Lymphomas with a Ki-67 index below 30% are categorized as indolent or low grade, suggesting a longer clinical course and less aggressive behavior [62]. This significant difference in proliferation rates helps pathologists differentiate during histological evaluations. It influences treatment choices, as regimens for high-grade lymphomas like Burkitt differ markedly from those for more indolent lymphoma types [16].

In diffuse large B-cell lymphoma (DLBCL) cases, Ki67 expression varies, with only a few exceeding 90%. It is crucial to evaluate this marker carefully to rule out non-tumoral cells.

**Figure 5:**
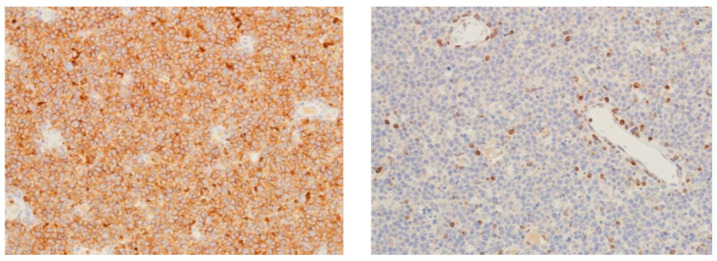
CD10 and BCL2 expression in Burkitt lymphoma 10×.

If cell size, CD10 expression, and Ki67 evaluation fail to differentiate BL from DLBCL, additional staining with BCL2, BCL6, and CD43 is recommended (Figure 5). Large B-cell lymphomas with plasmacytic, plasmablastic, or immunoblastic features should be assessed for CD138, ALK1, and HHV8 (LANA) expressions.

DLBCL rarely expresses CD138, whereas plasmablastic lymphoma often reacts positively for CD138, CD38, and MUM1/IRF4. This condition typically appears as lesions in the mouth or other non-lymphoid areas, particularly in individuals with HIV, weakened immune systems, or advanced age. Generally, these lymphomas lack CD45, PAX5, and CD20 markers. An ALK-1 immunostain should be performed on lymphomas with large cell morphology characterized by immunoblastic, plasmacytoid, or plasmablastic features in order to assess the likelihood of ALK-positive large B-cell lymphoma [63]. This rare neoplasm is distinguished by a unique intrasinusoidal nodal infiltration, similar to that observed in anaplastic large-cell lymphoma. ALK-positive large B-cell lymphomas generally express EMA, CD138, and MUM-1, but typically exhibit negative stain for CD30 and are predominantly negative or only weakly positive for B-cell markers.

Distinguishing plasmablastic lymphoma (PBL) from ALK-positive large B-cell lymphoma (ALK+ LBCL) is vital due to their overlapping morphologic and immunophenotypic features, yet different clinical behaviors and therapeutic implications. Both entities can present with a high proliferation index and may show plasmablastic characteristics; however, accurate diagnosis is essential to guide appropriate treatment decisions [64,65].

PBL is primarily associated with immunosuppressed conditions, such as HIV infection, and is characterized by a lack of typical B-cell markers (e.g., CD20, PAX5), while expressing plasma cell markers (e.g., CD138, MUM1, or IRF4) [66]. In contrast, ALK+ LBCL is a distinct entity classified under B-cell neoplasms, which often results from chromosomal aberrations involving the ALK gene, leading to aberrant expression of the ALK protein [47]. The treatment strategies differ significantly; while ALK+ LBCL may be responsive to ALK inhibitors, PBL typically resists conventional chemotherapy regimens due to its aggressive biological behavior [65].

The differentiation relies on a careful panel of immunostains. PBL is characteristically negative for CD20, whereas ALK+ LBCL typically retains this expression. The absence of CD20 in a suspected B-cell neoplasm strongly suggests PBL [66]. Plasma cell markers (CD138, MUM1/IRF4) are more likely to be expressed in PBL than in ALK+ LBCL, where B-cell markers dominate. The presence of CD138 and MUM1 helps reinforce a diagnosis of plasmablastic differentiation [64,67].

Immunostaining for the ALK protein is critical in identifying ALK+ LBCL. ALK positivity delineates this entity from PBL, which generally does not express this oncoprotein. Therefore, confirming ALK positivity in large B-cell lymphomas suggests an ALK-dependent pathogenesis rather than plasmablastic transformation [68,69].

Both conditions may show a high Ki-67 index; however, a significantly higher proliferation rate (often exceeding 90%) in PBL elevates suspicion for PBL rather than ALK+ LBCL, which may exhibit variations in proliferation rates [70].

In addition, the presence of plasmablastic morphology in large B-cell lymphoma necessitates testing for HHV8 to rule out HHV8 positivity DLBCL [71,72]. This extremely rare neoplasm may sometimes be linked to HHV8-positive Castleman disease and/or HIV infection.

In today’s fast-paced world of pathology, diagnosing DLBCL is mostly about spotting those large lymphoid cells and confirming their B-lineage through immunostaining. Over the last twenty years, we have made incredible strides in understanding the genetic quirks of large-cell lymphomas, which has helped us refine how we categorize large B-cell lymphomas. Because of this progress, pathologists find it more important than ever to uphold modern diagnostic accuracy standards when reviewing these cases.

This discussion aims to provide clear and practical guidelines to help navigate these sometimes tricky situations.

## 13. Conclusions

The diagnosis and classification of large B-cell lymphomas, particularly diffuse large B-cell lymphoma (DLBCL), remain complex yet critically important in modern hematopathology. The 2022 WHO Classification has introduced nuanced revisions that enhance diagnostic precision, guide therapeutic decisions, and reflect evolving insights into the genetic and phenotypic heterogeneity of these neoplasms. DLBCL, despite being the most common subtype, often presents diagnostic challenges due to its morphologic overlap with other aggressive lymphomas and nonhematopoietic malignancies.

Accurate subtyping of DLBCL relies on a multidisciplinary approach combining morphology, immunohistochemistry, cytogenetics, and molecular diagnostics. Key distinctions among germinal center B-cell-like (GCB), activated B-cell-like (ABC), and high-grade B-cell lymphomas, including double-hit and triple-hit variants, are critical for determining prognosis and tailoring treatment. The incorporation of diagnostic algorithms, such as the Hans classifier, and the judicious use of ancillary techniques like FISH and Ki-67 proliferation indexing are essential tools in the pathologist’s repertoire.

As lymphoma diagnostics continue to evolve, maintaining a high standard of diagnostic accuracy and an awareness of new classification criteria is imperative for improving patient outcomes.
